# Community perceptions and acceptability of mass drug administration for the control of neglected tropical diseases in Asia-Pacific countries: A systematic scoping review of qualitative research

**DOI:** 10.1371/journal.pntd.0010215

**Published:** 2022-03-11

**Authors:** Elke Mitchell, Angela Kelly-Hanku, Alison Krentel, Lucia Romani, Leanne J. Robinson, Susana Vaz Nery, John Kaldor, Andrew C. Steer, Stephen Bell

**Affiliations:** 1 Kirby Institute, UNSW Sydney, Sydney, Australia; 2 Papua New Guinea Institute of Medical Research, Goroka, Papua New Guinea; 3 Bruyère Research Institute, Ottawa, Canada; 4 School of Epidemiology and Public Health, University of Ottawa, Ottawa, Canada; 5 Tropical Diseases Group, Murdoch Children’s Research Institute, Melbourne, Australia; 6 Burnet Institute, Melbourne, Australia; 7 School of Public Health and Preventative Medicine, Monash University, Melbourne, Australia; 8 Department of Paediatrics, University of Melbourne, Melbourne, Australia; 9 Melbourne Children’s Global Health, Melbourne Children’s Campus, The Royal Children’s Hospital, Melbourne, Australia; 10 UQ Poche Centre for Indigenous Health, The University of Queensland, St Lucia, Australia; 11 School of Public Health, The University of Queensland, St Lucia, Australia; 12 Centre for Social Research in Health, UNSW Sydney, Sydney, Australia; University of Heidelberg, GERMANY

## Abstract

**Background:**

Preventative chemotherapy and mass drug administration have been identified as effective strategies for the prevention, treatment, control and elimination of several NTDs in the Asia-Pacific region. Qualitative research can provide in-depth insight into the social dynamics and processes underlying effective implementation of and adherence to mass drug administration programs. This scoping review examines published qualitative literature to examine factors influencing community perceptions and acceptability of mass drug administration approaches to control NTDs in the Asia-Pacific region.

**Methodology:**

Twenty-four peer reviewed published papers reporting qualitative data from community members and stakeholders engaged in the implementation of mass drug administration programs were identified as eligible for inclusion.

**Findings:**

This systematic scoping review presents available data from studies focussing on lymphatic filariasis, soil-transmitted helminths and scabies in eight national settings (India, Indonesia, Philippines, Bangladesh, Laos, American Samoa, Papua New Guinea, Fiji). The review highlights the profoundly social nature of individual, interpersonal and institutional influences on community perceptions of willingness to participate in mass drug administration programs for control of neglected tropical diseases (NTD). Future NTD research and control efforts would benefit from a stronger qualitative social science lens to mass drug administration implementation, a commitment to understanding and addressing the social and structural determinants of NTDs and NTD control in complex settings, and efforts to engage local communities as equal partners and experts in the co-design of mass drug administration and other efforts to prevent, treat, control and eliminate NTDs.

**Conclusion:**

For many countries in the Asia-Pacific region, the “low hanging fruit has been picked” in terms of where mass drug administration has worked and transmission has been stopped. The settings that remain–such as remote areas of Fiji and Papua New Guinea, or large, highly populated, multi-cultural urban settings in India and Indonesia–present huge challenges going forward.

## Author summary

Qualitative research can provide in-depth insight into the social dynamics and processes underlying effective implementation of and adherence to mass drug administration programs. This scoping review examines published qualitative literature to examine factors influencing community perceptions and acceptability of mass drug administration approaches to control neglected tropical diseases (NTDs) in the Asia-Pacific region. Our analyses highlight the profoundly social nature of individual, interpersonal and institutional influences on community perceptions of willingness to participate in mass drug administration programs for control of NTDs. For many countries in the Asia-Pacific region, the “low hanging fruit has been picked” in terms of where mass drug administration has worked and transmission has been stopped. The settings that remain–e.g. remote areas of Fiji and Papua New Guinea, or large, highly populated, multi-cultural urban settings in India and Indonesia–present huge challenges going forward. Future NTD research and control efforts would benefit from a stronger qualitative social science lens to mass drug administration implementation, a commitment to understanding the socio-structural determinants of NTDs and NTD control in complex settings, and engaging local communities as equal partners and experts in the co-design of mass drug administration and other efforts to prevent, treat, control and eliminate NTDs.

## Introduction

Neglected tropical diseases (NTDs) are a global public health and social issue, affecting more than one billion people worldwide [1]. Multiple NTDs–including soil transmitted helminths, scabies, lymphatic filariasis, schistosomiasis, trachoma and yaws–are endemic to many low- and middle-income countries (LMICs) in the Asia-Pacific region [2–5]. The burden of NTDs is most pronounced among low income populations in remote and rural areas, where access to health care is limited [6]. NTDs can cause serious health complications such as anaemia (soil-transmitted helminths), septicaemia (scabies), elephantiasis (lymphatic filariasis) and blindness (trachoma) and are responsible for 25 million disability adjusted life-years globally [7]. The social impacts of NTDs are profound, including reductions in economic productivity and educational attainment, and increasing experiences of stigma and discrimination [8,9]. Despite a growing body of research focusing on NTDs in LMICs, NTDs remain largely overlooked in national and global public health agendas [10].

Preventative chemotherapy and mass drug administration have been identified as effective strategies for the prevention, treatment, control and elimination of several NTDs in the Asia-Pacific region [11–13]. Preventative chemotherapy is the large-scale use of medicines with populations at risk of NTDs, either alone or in combination, in public health interventions [1]. Mass drug administration–one form of preventative chemotherapy–is the distribution of medicines to the entire population of a given setting, irrespective of the presence of symptoms or infection [1]. In Fiji, the use of ivermectin through mass drug administration reduced experiences of scabies from 32.1% to 1.9% in the study group [11]. In Indonesia, the use of diethylcarbamazine and albendazole through mass drug administration showed a drop in prevalence of microfilaremia from 26% to less than 1% [12].

Data on adherence during mass drug administration programs are mixed. In a study of lymphatic filariasis control in India, while 99% of study participants received tablets during the mass drug administration intervention, less than a third (28% in rural and 31% in urban areas) consumed the drugs [14]. In contrast, among 63.3% of the sampled population who received antifilarial drugs in a study in the Philippines, 94.5% ingested the drugs [15]. Common reasons for non-adherence during mass drug administration programs cited in quantitative surveys include fear of medication side effects, preference for other methods to treat illness, lack of awareness or understanding of mass drug administration programs and approaches, and other issues associated with drug distribution, such as being absent during time of drug distribution or a lack of training for drug distributors [16–20].

Qualitative research can provide in-depth insight into the social dynamics, processes and meanings underlying trends identified in survey-based and epidemiological research [21,22]. To date there has been limited insight from qualitative studies in NTD research and control efforts [23,24]. The transmission of NTDs, community attitudes towards programs that seek to control and eliminate NTDs, such as mass drug administration, and people’s willingness to be treated or consume tablets are influenced by a variety of socio-cultural, religious, political, economic and environmental contexts within any given community setting [25,26]. This paper reviews published qualitative literature–documenting primary data collected from community members and stakeholders involved in the implementation of mass drug administration programs–to examine the diverse social and contextual factors influencing community perceptions on and acceptability of mass drug administration approaches to control NTDs in the Asia-Pacific region.

### Systematic scoping review methodology

A systematic scoping review is a structured method used to synthesise and analyse published qualitative literature in a rigorous, transparent manner [27–31]. Scoping reviews typically address broad research questions, providing an overview and organisation of existing knowledge, rather than a narrow synthesis of a predefined research question [27,28,32]. Our aim was to undertake a comprehensive review of available published qualitative research on community and stakeholder perceptions and acceptability of mass drug administration and factors influencing adherence during mass drug administration programs in the Asia-Pacific region and identify current research gaps and future research priorities.

### Definitions and concepts

For the purpose of this manuscript, in line with the World Health Organisation [1], we defined NTDs as a diverse group of bacterial, parasitic, viral and fungal infections that persist in tropical and sub-tropical climate conditions and among populations living in poverty. This paper focuses on NTDs prevalent in LMICs in the Asia-Pacific region that have the potential to be effectively controlled using preventative chemotherapy. This included lymphatic filariasis, scabies, schistosomiasis, soil-transmitted helminths, trachoma and yaws. Preventative chemotherapy and mass drug administration are defined above. We also adopted the World Bank definition of low income, lower middle income and upper middle-income economies to define LMICs in the Asia-Pacific region [33].

### Identification of studies

The following databases were searched on 25 January 2021 to identify relevant papers: Medline, EMBASE, Global Health, Scopus, ProQuest, CINAHL, Emcare. These databases were searched using the following structure of search terms: [list NTDS] AND [list country settings] AND [list qualitative research methods]. Specific search terms used are detailed in Table 1. For the purpose of this paper, we included studies that used qualitative methods for data collection (i.e. interviews, focus groups, participant observation, ethnographic techniques, photovoice). We also included mixed methods studies that specifically stated that a qualitative study using these methods was included alongside quantitative data collection; only data from the qualitative components of mixed methods studies were included in this review.

**Table 1:** Search terms.

Thematic focus	Search terms	Add with:
NTD	Neglected tropical diseases OR NTDs OR neglected diseases OR lymphatic filariasis OR filariasis OR scabies OR *Sarcoptes scabiei* OR ectoparasites OR schistosomiasis OR bilharzia OR soil-transmitted helminthiases OR helminths OR hookworm OR roundworm OR whipworm OR trachoma OR yaws OR framboesia	AND
Setting	American Samoa OR Federated States of Micronesia OR Micronesia OR Fiji OR Kiribati OR Marshall Islands OR Nauru OR Papua New Guinea OR Samoa OR Solomon Islands OR Tonga OR Tuvalu OR Vanuatu OR Afghanistan OR Bangladesh OR Bhutan OR Cambodia OR China OR India OR Indonesia OR Lao PDR OR Malaysia OR Maldives OR Mongolia OR Myanmar OR Nepal OR Pakistan OR Philippines OR DPR of Korea OR Sri Lanka OR Thailand OR Timor-Leste OR East-Timor OR Vietnam	AND
Research method	Qualitative OR mixed methods OR interview OR focus group OR participant observation OR ethnograph* OR social science OR photovoice	AND

The results were limited to human studies from 2000 to 2020, reporting qualitative data collected with community members and/or stakeholders influencing the implementation of mass drug administration programs (i.e. policy makers, health workers, program implementation staff, teachers). Papers were excluded if the research was not published in English; the study sample or focus was outside LMICs in the Asia-Pacific region; reported exclusively on quantitative data; was not peer reviewed; and did not contain primary data. Unpublished grey literature, conference abstracts, conference reports and media articles were also excluded.

### Data extraction and synthesis

The final papers were reviewed using a data extraction tool designed by the authors for this scoping review. EM and SB independently reviewed each paper and conferred on which publications to include. Two types of information were documented. First, referencing information, study population, location of study, research methods and analysis procedures were collected. Second, data were extracted deductively [34] from each paper guided by a socio-ecological model of health promotion [35]. Socio-ecological models have been used analytically in health research to examine the diverse influences on people’s health seeking practices [36–39]. In this analysis, this socio-ecological model was used to examine diverse individual (e.g., knowledge, attitudes and practices), interpersonal (e.g., parents, family and community) and institutional (e.g., stakeholders, institutions, mass drug administration programs) factors influencing community perceptions of and adherence to mass drug administration programs to treat NTDs in the Asia-Pacific region. Further inductive analysis [34] was conducted to identify any unexpected themes within the published literature. The following findings section is structured around these deductive and inductive themes.

## Results

**Fig 1:**
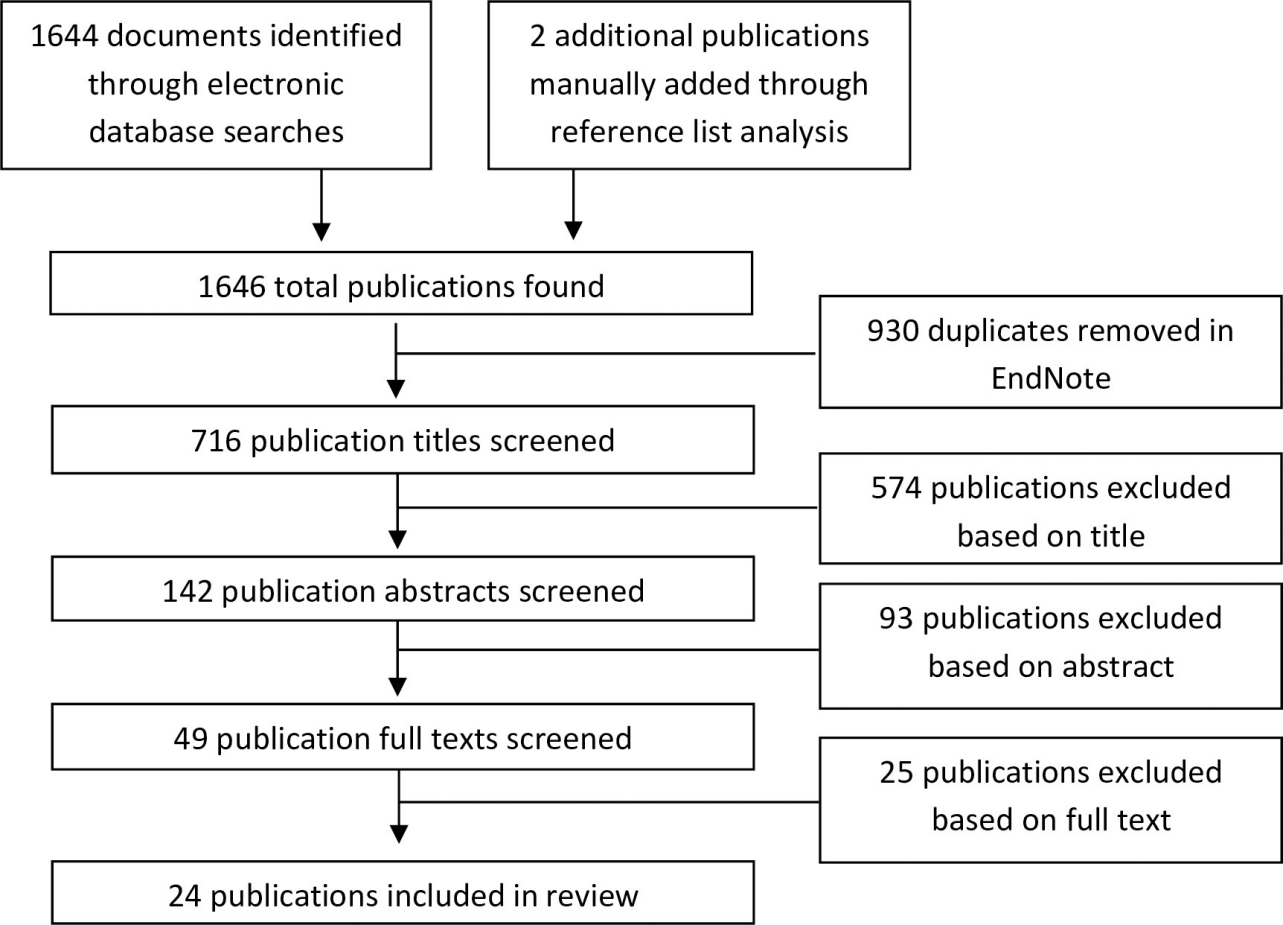
Flow chart of the selection process.

A total of 1646 unique references were identified, and after screening, 24 papers met the inclusion criteria for the scoping review (see Fig 1).

The characteristics of the final papers are summarised in Table 2. Collectively, these papers reported on data examining community and stakeholder perceptions of mass drug administration for lymphatic filariasis, soil-transmitted helminths and scabies control, as well as factors influencing adherence. Twenty papers reported on community perceptions, and 15 papers reported on stakeholder perceptions. Regarding study design, 11 papers reported on mixed methods studies, 11 papers reported on qualitative study designs, one paper reported on a rapid ethnographic study, and one paper reported on a study using open and closed survey questions.

**Table 2:** Summary of paper characteristics.

Citation	NTD	Research design	Study population	Location
***American Samoa***				
King et al. (2011)	Lymphatic filariasis	Mixed methods–surveys, interviews and focus groups	Community members: religious leaders. Stakeholders: nurses, program directors, health assistants, volunteer drug distributors.	American Samoa
***Bangladesh***				
Hafiz et al. (2015)	Soil-transmitted helminths	Mixed methods–survey and interviews	Stakeholders: STH control program staff, health officers, education officers, data collection personnel, teachers.	Munshiganj and Lakshmipur districts, Bangladesh
Nath, Padmawati & Murhandarwati (2019)	Soil-transmitted helminths	Mixed methods–survey, interviews and focus groups	Community members: community opinion leaders, school-aged children, parents Stakeholders: school teachers, mass drug administration program managers,	Urban and rural, Dhaka and Sylhet districts, Bangladesh
***Fiji***				
Mitchell et al. (2020)	Scabies	Interviews	Community members: adults	Vanua Levu, Fiji
***India***				
Ramaiah et al. (2000)	Lymphatic filariasis	Mixed methods–Survey, interviews and focus groups	Stakeholders: ‘key informants’, medical officers, health workers.	Tamil Nadu, India
Ramaiah et al. (2001)	Lymphatic filariasis	Mixed methods–Survey, interviews and focus groups	Community members: adults Stakeholders: ‘key informants’, medical officers, health workers, community drug distributors.	Tamil Nadu, India
Babu and Kar (2004)	Lymphatic filariasis	Mixed methods–survey, interviews and focus groups	Community members: adults, filariasis patients Stakeholders: NGO workers, private practitioners, medical officers, district level policy makers, health workers	Urban and rural, Orissa, India
Babu and Nath (2004)	Lymphatic filariasis	Interviews and focus groups	Community members: adults Stakeholders: medical officers, private practitioners, health workers, NGO staff.	Orissa, India
Babu and Mishra (2008)	Lymphatic filariasis	Mixed methods–survey and interviews	Community members: heads of households (mix of compliant and non-compliant households from urban and rural areas)	Urban and rural, Orissa, India
Lahariya and Mishra (2008)	Lymphatic filariasis	Mid-term evaluation–interviews and observation	Community members: adults Stakeholders: ‘key informants’	Urban and rural, Madhya Pradesh, India
Babu (2010)	Lymphatic filariasis	Interviews and focus groups	Community members: adults Stakeholders: private practitioners, medical officers, district level policy makers, health workers, community drug distributors, mass drug administration program staff (incl. doctors).	Urban and rural, Orissa, India
Hussain et al. (2014)	Lymphatic filariasis	Survey–open- and close-ended questions	Community members: adults Stakeholders: medical officers, drug distributors, health workers.	Puri district, Bay of Bengal, India
Banerjee et al. (2019)	Lymphatic filariasis	Mixed methods–survey and interviews	Community members: adults	Urban, Nagpur City, India
Nandha et al. (2019)	Lymphatic filariasis	Mixed methods–Survey, interviews and focus groups	Community members: adults Stakeholders: ‘key informants’	Palakkad, India
Aruldas et al. (2020)	Soil-transmitted helminth	Focus groups	Community members: adults	Tamil Nadu, India
***Indonesia***				
Krental and Aunger (2012)	Lymphatic filariasis	Interviews	Community members: adults (equal balance of women and men, compliers and non-compliers, urban and rural areas)	Urban and rural, Alor district, Indonesia
Ikawati, Wijayanti and Jastal (2018)	Lymphatic filariasis	Interviews	Community members: village leaders	Pasaman Barat District, Indonesia
Krentel and Wellings (2018)	Lymphatic filariasis	Interviews	Community members: adults (equal balance of women and men, compliers and non-compliers, urban and rural areas)	Urban and rural, Alor district, Indonesia
***Papua New Guinea***				
Wynd et al. (2007)	Lymphatic filariasis	Interviews and focus groups	Community members: adults Stakeholders: ‘key informants’	Rural, Milne Bay Province, Papua New Guinea
***Philippines***				
Amarillo et al. (2008)	Lymphatic filariasis	Mixed methods–survey, interviews and focus groups	Community members: community leaders Stakeholders: local health officers, field health personnel	Agusan del Sur, Philippines
Bacon et al. (2012)	Soil-transmitted helminths	Mixed methods–survey and interviews	Community members: parents Stakeholders: teachers	Provinces of Capiz, Antique and Aklan, Philippines
Labana et al. (2019)	Soil-transmitted helminths	Interviews, focus groups and observation	Stakeholders: teachers, nurses, dentist	Cagayan, Philippines
Lorenzo et al. (2019)	Soil-transmitted helminths	Focus groups	Community members: parents, children	Rural, Provinces of Northern Samar and Sorsogon, Philippines
***Lao PDR***				
Bardosh et al. (2014)	Soil-transmitted helminths	Rapid ethnography–participant observation, unstructured and semi-structed interviews and focus groups	Community members: adults Stakeholders: ‘key informants’	Remote, Lao PDR

Papers reporting on studies from India [14,40–49], Indonesia [50–52], Philippines [15,53–55], Bangladesh [19,56], Laos [57], American Samoa [18], Papua New Guinea [58] and Fiji [25].

Sixteen studies focused on lymphatic filariasis. This included six mixed methods studies, three qualitative studies and one study using open and closed survey questions in India [14,40–48]; three qualitative studies conducted in Indonesia [50–52]; one mixed methods study in the Philippines [15]; one mixed methods study in American Samoa [18]; and one qualitative study in Papua New Guinea [58]. Seven studies focused on soil-transmitted helminths. These included one mixed methods study and two qualitative studies in the Philippines [53–55]; two mixed methods studies in Bangladesh [19,56]; one qualitative study in India [49]; and one rapid ethnography in Laos [57]. One qualitative study focused on scabies in Fiji [25].

### Individual influences

#### Perceptions of mass drug administration programs

Perceptions of mass drug administration approaches and knowledge of the potential health benefits of participation were mixed. Eight studies reported positive community perceptions of mass drug administration programs, including perceived and actual experiences of the benefits of ingesting medication such as a decrease or elimination of parasites from the body, preventing and interrupting transmission, reduced healthcare costs and continued good health [25,41,42,47–49,52,58]. In two studies [41,55], participants reported that experiencing adverse reactions after ingesting medications such as vomiting, dizziness or the passing of worms from the body was a sign the drugs were effective and the program had been successful.

Misconceptions about mass drug administration programs to treat NTDs were noted among community members in ten studies [14,25,40,41,44,47,49,50,52,58]. Participation in programs was considered unnecessary due to a belief that good health, the absence of disease in the family or community and the absence of symptoms such as passing worms or swollen legs meant ingesting medication was needless, resulting in lowered adherence rates [14,25,40,41,44,47,49,50,52,58]. Participation in the program was only considered necessary for people infected with the disease, demonstrating limited understanding of the whole of population approach required to eliminate NTDs using mass drug administration approaches. Other misconceptions included the belief that participation in previous lymphatic filariasis mass drug administration programs was thought to have provided ‘long-term immunity’ in one study in Papua New Guinea [58].

#### Fear of adverse side effects

Fear of adverse side effects from ingesting drugs provided during mass drug administration programs was reported as common among community members in a wide range of studies examining lymphatic filariasis [14,15,41,43,44,47,52,58], soil-transmitted helminths [19,49,55] and scabies [25]. Personal or interpersonal experiences of side effects such as nausea, fever, headaches, rash and vomiting during current or previous mass drug administration programs increased fear and unwillingness to ingest medication [14,15,19,41,43,44,47,52,55]. One paper in Nagpur, India noted that participants experiencing chronic health conditions such as hypertension and diabetes, or those who had recently undergone surgery, refused to comply to a program [44]. This was due to fear that consuming drugs administered during a mass drug administration program alongside their current drug regime may result in adverse drug interaction and negative side effects [44]. Similar concerns were raised in studies in Vanua Levu, Fiji [25] and Tamil Nadu, India [49].

### Interpersonal influences

#### Family and community endorsement of mass drug administration programs

Social influences within families and communities were reported as impacting adherence rates during mass drug administration programs. Within households, family dynamics and beliefs around the benefits of mass drug administration in reducing risk of lymphatic filariasis were noted in two studies [41,51]. Men’s power and responsibility as head of household was reported as a key factor influencing access, uptake and adherence of mass drug administration in a study in Indonesia [51]. Observation of unwanted side effects of drugs among family members discouraged adherence in another study in Indonesia [52].

In four studies, endorsement of programs by village leaders, community acceptance of mass drug administration approaches, knowledge of neighbours’ adherence or non-adherence during programs, and norms of adherence with government-led programs more generally were reported as influencing people’s participation in mass drug administration programs [41,46,50,52]. Rumours within the community and newspaper reports of people becoming seriously ill and even dying after consuming medication was reported as reducing adherence rates during lymphatic filariasis mass drug administration programs in India [14,40,43,45].

#### Parental attitudes and beliefs

The beliefs and attitudes of parents were noted as inhibiting the success of school-based mass drug administration programs for soil-transmitted helminths. Parental refusal to provide consent to children’s participation inhibited the reach of school-based programs in four studies [19,53–55]. Reasons for unwillingness to provide consent included in these papers were a lack of perceived need for deworming; a lack of trust in the drugs provided during programs; a preference for traditional disease treatment; concerns over erratic worm migration; children being sick or feverish during programs; the potential for adverse side effects; and concerns over limited training of teachers to handle these adverse reactions [19,53–55].

#### Cultural frameworks of understanding illness and pharmacology

Local beliefs relating to illness and pharmacology that are shared and communicated through interpersonal relationships were possible barriers to adherence during mass drug administration programs in four studies. In Papua New Guinea and Fiji, the use of local herbs was viewed as a way to treat or heal lymphatic filariasis [58] and scabies [25]. In Laos, the potential for poor adherence in future mass drug administration programs due to a belief that consuming medicines when not unwell will cause them to become ‘stored’ resulting in a build-up on ‘toxins’ in the body, was noted [57]. In a study in Indonesia, some interviewees felt that their use of traditional medicine meant that they were protected from lymphatic filariasis, and therefore did not need to take the drugs associated with the mass drug administration program [52].

### Institutional influences

#### Informational support and community consultation

Eight papers identified that a lack of program information and community consultation *before* programs commenced inhibited community understanding, acceptability and participation in mass drug administration programs [14,15,19,40,41,44,45,47]. In several studies, community members reported that communities and households were not adequately informed about the program, including the date and time of drug distribution [15,40,41,44,45,47]. The lack of specific information about mass drug administration programs in lymphatic filariasis educational materials and a failure to translate messaging into local dialects were identified as program shortcomings in the Philippines [15]. Several papers identified the need for improved pre-program information and advocacy to address concerns around adverse side effects, improve knowledge of the benefits of this NTD control approach and improve adherence rates [19,43,54].

In contrast, comprehensive provision of information and community consultation prior to drug distribution was attributed to increased coverage and adherence in three studies [40,54,57]. Two studies reported how community consultation–in the form of regular community engagement and meetings where detailed information was provided–increased community members’ willingness to participate in mass drug administration programs [40,57]. In one study about a school-based program in the Philippines, teachers were credited with helping to facilitate the program though organising a meeting with parents to provide information on the rationale for mass drug administration, the approach and possible side effects of treatment, and used the meetings as an opportunity to distribute and collect consent forms [54]. One study in American Samoa identified church leaders as a possible medium through which information and advocacy could be disseminated to community members prior to the program commencing [18].

Five papers reported the importance of informational support provided by community groups, health workers and drug distributors *during* and *after* mass drug administration programs in increasing adherence rates [15,40,41,43,46]. Three papers noted that a lack of information provision during drug distribution lowered adherence rates in studies in Orissa and Nagpur, India [40,41,44]. Community members’ perceived acceptability of mass drug administration programs was enhanced by health workers and drug distributors taking the time to explain the rationale for these approaches, the management of adverse side effects, clarifying misconceptions about the drugs administered, and follow up after the program [15,41,43,46]. Active roles for community groups (e.g. women’s groups, youth groups) during programs–in the form of distributing information and sharing experiences–was noted as increasing both coverage and adherence rates in two studies in India [40,46].

#### Stakeholder knowledge and attitudes

Attitudes and beliefs about mass drug administration among stakeholders including medical officers, private practitioners, health workers, non-government organisation personnel and drug distributors, were mixed. Good technical knowledge of and clear rationale for this approach, and a positive perception of mass drug administration in eliminating disease, were noted as positive influences on implementation of these programs in Orissa and Tamil Nadu, India [42,47,48,59]. In contrast, negative perceptions and poor knowledge of mass drug administration were reported among stakeholders in India and Papua New Guinea [42,58,59]. A study of clinician attitudes towards mass drug administration to control lymphatic filariasis in India noted limited knowledge of scientific rationale of this approach, disbelief that a single-dose approach would be effective, and that lifestyle changes alongside vector control were necessary for elimination [59]. In another study of stakeholders (i.e. medical officers, private practitioners, health workers, non-government organisation personnel) in India, negative perceptions of mass drug administration to manage lymphatic filariasis included perceptions that these programs used poor-quality drugs; adverse side effects were poorly managed during programs; mosquito control was the only effective way to prevent lymphatic filariasis; and an apparent lack of understanding of the rationale or benefits of mass drug administration [42].

#### Training for health workers and drug distributors

A lack of adequate training of key stakeholders involved in delivering mass drug administration programs, including health workers and drug distributors, inhibited the success of programs in five studies [14,19,45,46,53]. In one study of a school-based mass drug administration program in Bangladesh, school teachers expressed concern they had not received any training and lacked knowledge about soil-transmitted helminths, preventative management and the drugs to be distributed, and were not equipped to answer potential questions posed by students and parents [19]. In Bangladesh and the Philippines, school teachers and health workers were concerned that they would be blamed by parents if children experienced adverse side effects from ingesting medication [19,53]. In contrast, one study in India noted high adherence rates in locations where training of health staff involved in the mass drug administration programs had taken place [40].

#### Practices of administering drugs

Reluctance to consume drugs administered during these programs was identified in studies in India, Philippines and Papua New Guinea [40,41,44,47,49,55,58]. In some cases, community members were suspicious or confused when provided with loose tablets without labels or instructions [40,41,44]. Others were apprehensive about the large number of tablets they were asked to consume [44,47,58]. Three studies identified a lack of trust in the quality and effectiveness of drugs supplied freely by the government [44,49,55], with community members in one study opting instead to purchase medication from pharmacies or use home remedies [55]. In India, refusal to take medication that was not supplied by a known doctor was reported as a barrier to adherence [44].

Community involvement in drug distribution–including through village birth attendants, community-based health workers and teachers–was reported as an effective way to dispense drugs in a study in Papua New Guinea [58]. However, use of ‘community-directed treatment’ (i.e. community-led treatment distribution) in a study in India reported challenges to communities taking responsibility for the distribution of drugs [48]. Barriers to community members acting as drug distributors included a lack of commitment to implement the program from community leaders, community members’ hesitancy to accept drugs from community drug distributors, and group and caste conflicts within some villages.

A range of process issues were identified as impeding coverage and adherence rates in mass drug administration programs. These included delays in the supply of drugs, and consequential interruption to program implementation [40,45,54]; an absence of community members during specific times when drugs were distributed due to work commitments and seasonal and labour migration, and lack of repeat visits for drug distribution [44,45,49,57]; a lack of drug distributors and time constraints [47]; a lack of strategy to reach children out of school in school-based programs [19,56]; and limited effort to monitor drug ingestion in children [19].

## Discussion

Findings from this scoping review highlight the importance of qualitative evidence to optimise delivery of mass drug administration programs in diverse socio-cultural settings, and the value in gathering further country-specific qualitative perspectives to maximise local impact of future programs across the Asia-Pacific region. Our analysis points to specific knowledge gaps. First, papers that were reviewed focused only on three NTDs–lymphatic filariasis, soil-transmitted helminths and scabies. There is an absence of published qualitative research to inform effective control of other NTDs prevalent in LMICs in the Asia-Pacific region–i.e. schistosomiasis, trachoma, yaws–using preventative chemotherapy. Second, only three published papers examined NTDs (on lymphatic filariasis and scabies) in the Pacific, creating a dearth of qualitative evidence from which to guide implementation of effective NTD control programs in the Pacific region more specifically. Our analysis contributes to the growing body of NTD-related systematic, rapid and scoping reviews with a focus on, for example, social stigma [60]; treatment adherence [61,62]; community understandings of mass drug administration for specific NTDs [63]; the role of nurses, community health workers and community drug distributors in responses to NTDs [64] [65,66]; and innovation in responses to tackling NTDs [67–72].

Beyond this, our scoping review reveals the profoundly social nature of mass drug administration approaches to NTD control in international settings in the Asia-Pacific region. We highlight diverse, locally-situated, individual, interpersonal and institutional influences on community perceptions of, adherence to and coverage of mass drug administration approaches for lymphatic filariasis, soil-transmitted helminths and scabies control. For instance, at an individual level, our analysis noted community members’ confusion and misconceptions about the need to participate in mass drug administration approaches to control NTDs [14,25,40,41,44,47,49,50,58], and fear of adverse side effects of ingesting drugs [14,15,19,25,41,43,44,47,49,52,55,58], which affected adherence rates and coverage in these settings. Both concerns are relational, arising from outcomes of communication between people, in households or communities, or between community members and health professionals. These scoping review findings point to the possible benefits of exploring how communication about mass drug administration programs and the importance of high coverage–in culturally appropriate language, using community relevant communication practices through locally trusted networks–might enhance the aims of NTD control and elimination at a population level.

At an interpersonal level, perceptions of mass drug administration programs and views on adherence were socially located in relations of power, control and word of mouth in community institutions. For instance, family endorsement of mass drug administration programs [19,41,51,53–55] is bound up in gendered decision making processes related to family health, which tends to rest primarily with men as heads of the household [51]. In schools, the power to enable children to participate in programs to treat for soil-transmitted helminths is situated in the hands of parents [19,53–55]. In turn, parents can be influenced by community attitudes about taking drugs administered during programs [14,40,41,43,45,46,50], which are produced by observing adherence practices of neighbours and other community members, or rumours of serious illness or death following drug ingestion [14,40,41,43,45,46,50]. Trust associated with longstanding cultural frameworks for understanding illness and pharmacology also had a powerful influence on potential adherence during mass drug administration programs [25,52,57,58]. Such findings demonstrate the influence of locally situated social relations and norms on individual and collective practices related to mass drug administration adherence, that reach far beyond biomedical and public health approaches to disease control based around taking tablets.

At an institutional level, where efforts to work respectfully and consultatively with community members in mass drug administration processes were limited or lacking, coverage and adherence rates suffered [14,15,19,40,41,44,45,47,49,54,55,57,58]. Identified process constraints included an absence of informational support and community consultation before, during and after mass drug administration programs; information on intervention logistics; culturally appropriate educational materials; and drug distributors making repeat visits to account for people’s daily and seasonal responsibilities. Other concerns related to the quality of drugs, the number of tablets to be ingested, and delays to drug supply. In contrast, when health workers and drug distributors took the time to explain the rationale for mass drug administration programs, discuss how to identify and manage adverse side effects and provide follow up visits after treatment, community members’ satisfaction with mass drug administration improved [15,41,43,46]. Such findings highlight the potential for a central role for communities in the design and delivery of future mass drug administration programs. This is not just in terms of being involved in distributing information or drugs, or implementing programs that are conceived of and designed elsewhere, or imposed on communities by others. Instead it is about drawing on people’s local knowledges and expertise–of families and community; of social networks and relations; of languages and communication practices; of social relations, values, belief systems and norms–from the point at which a mass drug administration program is being conceived in any social setting.

### Limitations

There are some limitations to this scoping review. We report on 24 papers, which were limited to studies published in peer reviewed journals. We excluded other potential sources of information including documents not published in English and grey literature in the form of government and community reports. Consistent with the systematic scoping review methodology, we did not assess the quality of the research presented in the papers reviewed, but instead identified and summarised key themes; the analyses presented from a scoping review are used to identify gaps in knowledge about issues under investigation, rather than to assess transferability of findings from particular qualitative studies beyond the settings in which the research was conducted. Despite these limitations, this scoping review of qualitative literature has identified important evidence and knowledge gaps that may help to improve the design and delivery of future mass drug administration programs to prevent and treat NTDs in the Asia-Pacific region.

### Future research

There is continued neglect of qualitatively informed social science perspectives in NTD research and control efforts. This in turn, inhibits best-practice design of programs and strategies that consider and engage with the social, cultural, religious, economic and environmental determinants of these diseases [23,24]. The result is a bias towards biomedical mechanisms to control and eliminate NTDs, without benefitting from the expertise gained from locally tailored and led, community-centred approaches that pay attention to relationships of power, trust and knowledge production in many global settings [23,24,73]. This is compounded by other global processes which include the longstanding problem of global health agendas being driven by richer countries in the global North, a narrow conceptual focus on therapeutic responses to illness and disease, and the preferred use of methodologies in public health that aim to achieve generalizability rather than attention to social context and local specificity [74–76]. Mass drug administration for NTD control and elimination will only reduce the prevalence of disease if significant proportions of the target population comply with programs. This means asking a lot of people to take a pill (or more than one pill), which, as a practice, is locally situated in social, cultural, religious, institutional and historical contexts [52]. Rigorous qualitative research can shed much needed light on the socio-structural factors that influence effective implementation of mass drug administration programs for maximum adherence and coverage, and that can also influence the success of NTD prevention, control and elimination efforts.

Our analysis identifies priorities for future qualitative research. First, a ‘social public health approach’ [77,78] to the prevention, control and elimination of NTDs would bring focus on the social dimensions of biomedical programs such as mass drug administration. This requires an understanding of the social relations and individual and collective practices and actions in communities that inhibit and enhance health and wellbeing, and drive change.

Second, research that explores how to engage the local skills, expertise and knowledges of community members to work alongside health professionals and epidemiologists in the co-design and delivery of context specific, tailored mass drug administration programs would help to ensure that NTD responses better fit the health needs and value systems of a given community or setting [23,73]. Consideration might also be given on how to avoid ‘top down’ approaches that overlook socio-cultural and political contexts of power, while ensuring stakeholders involved in mass drug administration delivery are well-versed in the contexts in which programs are delivered [23,26,73].

Third, interdisciplinary research–qualitative and quantitative social science, epidemiology and biomedical approaches–that is committed to addressing the root causes of NTDs, such as poverty, inequity, and political and environmental contexts, rather than focusing on biomedical technologies alone, must also occur if NTD prevention, treatment, control and elimination efforts are to be effective [23,24,26]. This is in line with WHO’s new road map for NTDs 2021–2030 [1].

## Conclusion

Our findings highlight the profoundly social nature of individual, interpersonal and institutional influences on community perceptions of willingness to participate in mass drug administration programs. For many countries in the Asia-Pacific region, the “low hanging fruit has been picked” in terms of where mass drug administration has worked and transmission has been stopped. The settings that remain–such as remote areas of Fiji and Papua New Guinea, or large, highly populated, multi-cultural urban settings in India and Indonesia–present huge challenges going forward. Future NTD research and control efforts would benefit from a stronger qualitative social science lens to mass drug administration implementation, a commitment to understanding and addressing the social and structural determinants of NTDs and NTD control in complex settings, and efforts to engage local communities as equal partners and experts in the co-design of mass drug administration and other efforts to prevent, treat, control and eliminate NTDs.
